# High-resolution melting curve analysis for infectious bronchitis virus strain differentiation

**DOI:** 10.14202/vetworld.2020.400-406

**Published:** 2020-03-03

**Authors:** Mustafa Ababneh, Ola Ababneh, Mohammad Borhan Al-Zghoul

**Affiliations:** Department of Basic Medical Veterinary Sciences, Jordan University of Science and Technology, Irbid 22110, Jordan

**Keywords:** high-resolution melting curve analysis, infectious bronchitis virus, spike 1 gene

## Abstract

**Background and Aim:**

Belonging to the *Coronaviridae* family, avian infectious bronchitis virus (IBV) causes respiratory, reproductive, and renal diseases in poultry. Preventative measures lie mainly in vaccination, while the gold standard for IBV classification and differentiation is based on the sequence analysis of the spike 1 (S1) gene. In this study, we tested a new assay for IBV strain classification that is less expensive and requires reduced time and effort to perform. We carried out a quantitative real-time polymerase chain reaction followed by high-resolution melting (qRT-PCR/HRM) curve analysis.

**Materials and Methods:**

In this study, qRT-PCR was conducted on a partial fragment S1 gene followed by a high resolution melting curve analysis (qRT-PCR/HRM) on 23 IBV-positive samples in Jordan. For this assay, we utilized the most common IBV vaccine strains (Mass and 4/91) as a reference in the HRM assay. To evaluate the discrimination power of the qRT-PCR/HRM, we did the sequencing of the partial S1 gene.

**Results:**

It was shown that HRM was able to classify IBV samples into four clusters based on the degree of similarity between their melting points: The first cluster exhibited the highest similarity to the 4/91 strain, while the second was similar to the Mass-related IBV strain. Although the third cluster contained the highest number of samples, it displayed no similarity to any of the reference vaccine strains, and, after comparing them with the sequencing results, we found that the samples in the third cluster were similar to the variant II-like (IS-1494-06) IBV field strain. Finally, the fourth cluster comprised one unique sample that was found to belong to the Q1 IBV strain.

**Conclusion:**

Our developed qRT-PCR/HRM curve analysis was able to detect and rapidly identify novel and vaccine-related IBV strains as confirmed by S1 gene nucleotide sequences, making it a rapid and cost-effective tool.

## Introduction

Avian infectious bronchitis (IB) serves as a major threat to the worldwide poultry industry, and it is a disease endemic to Jordan, causing respiratory, renal, and reproductive problems in chickens [1,2]. IB virus (IBV) is a positive-sense single-stranded RNA virus, belonging to the Coronavirinae subfamily gamma-coronaviruses [3]. IBV was first recognized as an avian respiratory pathogen in 1930, and its genetic diversity has resulted in the introduction of many IBV vaccines [4]. Recently, emerging IBV variants have been reported to cause nephropathogenic and reproductive problems, which are a turn of events that require a dramatic change in vaccination programs [5,6].

The IBV genome has an approximate size of 27,500 nucleotides and is organized in the following order: UTR5’-POl-S-3a-3b-E-M-4b-4c-5a-5b-N-UTR3’ [7]. The spike glycoprotein gene, encoding spike protein subunit 1 (S1) and 2 (S2), is the most variable gene in the IBV genome [8,9]. In particular, the S1 protein, which is responsible for viral attachment and entry into host cells, differs significantly with regard to amino acid sequence among IBV serotypes [10]. This variability makes the S1 gene an ideal target in viral typing assays incorporating reverse transcription-polymerase chain reaction (PCR) and DNA sequencing techniques. In most cases, high S1 gene homology between two strains can predict the occurrence of cross-protective immunity, where exposure to one strain confers protection to another [10-12].

IBV strains can be classified into six genotypes that together comprise 32 distinct viral lineages, the majority of which belong to genotype 1 [13]. In the Middle East, the common circulating IBV strains are the Massachusetts (Mass) and H120 vaccine strains as well as the D274, IBV variant I (793/B, IS/222/96, IS/251/96, and IS/64714/96) and variant II strains (IS/223/96, IS/572/98, IS/585/98, and IS/589/98) [1]. By the end of 2009, variant II and variant II-like strains appeared in Jordan (unpublished data) and North Iraq, and, in 2011, the unique IBV Q1 strain was reported in Jordan, Iraq, and Saudi Arabia [14]. For clinical purposes, IBV is diagnosed using RT-PCR and quantitative real-time PCR (qRT-PCR), but, for strain identification, sequencing of the full S1 gene or one of its fragments is performed [13,15].

High-resolution melting (HRM) curve analysis is a newly established PCR-based technique that has been used to differentiate related strains of the same animal or avian virus [16-19]. This technique was performed on double-stranded DNA resulting in rapid genotyping of genetic polymorphisms in diagnostic and routine [20]. This study aimed to optimize an HRM assay for genotyping the IBV vaccine and field strains found in Jordan.

## Materials and Methods

### Ethical approval

Approval from the Jordan University of science and technology animal care and use committee (JUST-ACUC) is not applicable as these samples were collected from recently dead chickens in private farms, and then samples were submitted to our laboratory.

### Vaccine and field strains

IBV vaccines were purchased from local Jordanian markets. The most commonly used IBV vaccine strains in Jordan are Mass-type strains (M41, MA5, and H120) as well as the 4/91 (793/B) vaccine strain (Table-1). These vaccines were used as reference IBV strains in the qRT-PCR/HRM procedure. Twenty-three positive IBV multi-tissue samples collected from different regions of Jordan during various outbreaks were also used (Table-2). Viral RNA of these samples and IBV vaccine strains were extracted, then went through cDNA synthesis, and then nested PCR amplification of a spike 1 gene fragment. To differentiate between IBV strains, the PCR products from the second round of nested S1 gene PCR were subjected to HRM analysis on the Rotor-Gene Q 5plex HRM Platform (Qiagen, CA, USA).

**Table-1:** The IBV vaccine strains used as a reference in this study.

Vaccine ID	Commercial name	IBV strain
1V	IB Mass	Mass 41
2V	IB 4/91	4/91 (793/B)
3V	IB 88	CR 88 variant strain (close to 793/B)
4V	CEVAC I BIRD	1\96 (close to 793/B)
5V	Cevac Brone 120 l	H 120

IB: Infectious bronchitis, IBV: IB virus

**Table-2:** The IBV field isolates used in this study.

Number	Sample ID	Tissue type
1	T1	Trachea
2	T2	Trachea
3	T3	Trachea
4	T4	Trachea
5	T5	Trachea
6	L1	Lung
7	L2	Lung
8	K1	Kidney
9	K2	Kidney
10	20417	Unknown
11	21417	Unknown
12	22417	Unknown
13	51216D	Trachea and cecal tonsils
14	61216D	Cecal tonsils
15	71216D	Cecal tonsils and kidney
16	101216D	Kidney
17	6417P	Lung and trachea
18	7417P	Lung and trachea
19	8417P	Lung and trachea
20	010217P	Lung and trachea
21	050617P	Allantoic fluid
22	D10117	Brain
23	7P6	Unknown

### RNA extraction and cDNA synthesis

The IBV vaccine vials were rehydrated with 1 ml of 1× PBS, then mixed by vortexing to dissolve the vaccines. A 150 µl of the rehydrated vaccine was added to 250 µl of TRI Reagent (Zymo Research, USA) and suspended for 5 min in a 50°C dry bath. Then, 400 µl of absolute ethanol was added, and RNA extraction was performed using the Direct-zol RNA MiniPrep kit (Zymo Research, USA). IBV field samples, mostly consisting of tracheal and kidney tissues that were submitted to our laboratory for IBV diagnosis, were homogenized and centrifuged at 3000× *g* for 5 min. After that, 150 µl of the supernatant was mixed with 250 µl of Trizol. A SuperScript^®^ IV First-Strand Synthesis kit (Invitrogen, USA) was used according to the manufacturer’s instructions to make cDNA for both the vaccine and field samples.

### Nested RT-PCR amplification of spike gene

Nested RT-PCR amplification was performed using the synthesized cDNA to amplify S1 gene fragments of the IBV genome. The first and second rounds nested PCR was performed in a reaction volume of 25 µl that contained 2.5 µl of 10× PCR buffer without magnesium, 1 µl dNTPs, 1 µl of each reverse and forward primers, 0.75 µl MgCl_2_, 0.1 µl *Taq* DNA polymerase, 1 µl cDNA, and 18.15 µl H_2_O. Thirty-five cycles of amplification were carried out in a thermocycler, with each cycle consisting of denaturation for 45 s at 94°C, annealing for 1 min at 50°C (for both rounds), and extension for 2 min at 72°C, and a final extension for 7 min at 72°C. Electrophoresis of amplified products was carried out using 1.5% agarose gel, which was visualized and photographed under UV light with a 100 bp ladder. Nested PCR primer sequences for the S1 gene were as previously reported [14].

### QRT-PCR/HRM

For this assay, the nested RT-PCR for the amplification of S1 fragment was repeated. However, the S1 RT-PCR products from the first round of nested RT-PCR were used as a template in the HRM protocol. A Type-it HRM PCR Kit (Qiagen, CA, USA) was employed according to the manufacturer’s instructions on the Qiagen Rotor-Gene Q 5plex HRM Platform (Qiagen, CA, USA). The qRT-PCR 25 µl reaction volume contained 12.5 µl of 2× HRM PCR master mix, 1.75 µl of each 10 µM S1 gene internal primers for the second round, and 2 µl of S1 RT-PCR product from the first round of nested PCR. The optimized cycling protocol for HRM analysis on the Qiagen Rotor-Gene Q 5plex HRM consisted of the following conditions: An initial PCR activation step for 5 min at 95°C followed by 40 cycles involving denaturation for 10 s at 95°C, annealing for 30 s at 55°C, and extension for 10 s at 72°C. HRM is for 2 s from 65 to 95°C with 0.1, 0.2, and 0.3°C increments.

### HRM analysis

Two softwares were used for the analysis of the HRM results. The operating HRM software installed on the Rotor-Gene Q 5plex machine and the Rotor-Gene ScreenClust HRM software (Qiagen, CA, USA) was used to analyze the results of HRM and to assign the different IBV strains into clusters according to their HRM patterns. The HRM analysis using the operating software was done by setting a reference IBV genotype either as 4/91, Mass, or variant II genotypes, after normalizations, the HRM genotype confidence percentages (GCPs) were obtained. For the ScreenClust software, the unsupervised model was used and then data were normalized and clusters were generated to contain all IBV isolates. The typicality and probability of each sample was recorded.

### Sequencing of partial S1 fragments and sequence analysis

The S1 RT-PCR products of the nested RT-PCR amplification were treated with ExoSAP-IT PCR Product Cleanup Reagent (Cat. No: 78201, Thermo Fisher Scientific, USA) according to the manufacturer’s instructions. Briefly, 2 µl of ExoSAP enzyme were added to 5 µl of the product then incubated at 37°C for 15 min to degrade remaining primers and nucleotides followed by another incubation at 80°C for 15 min to inactivate the ExoSAP-IT reagent. The cleaned RT-PCR products were sent to a sequencing facility (Macrogen, South Korea) to be sequenced by chain termination technology. S1 sequences were viewed on the BioEdit software and edited by the Edit Sequence interface of the Lasergene package. Sequence alignment, calculation of the sequence nucleotide similarities, and construction of the phylogenetic tree were done by the MegAlign interface of the Lasergene package. The partial sequences of S1 gene of all IBV isolates included in this study were deposited in GenBank under the accession numbers; MK680008-MK6800034.

## Results

### Molecular characterization (QRT-PCR/HRM and partial S1 sequencing)

**Figure-1:**
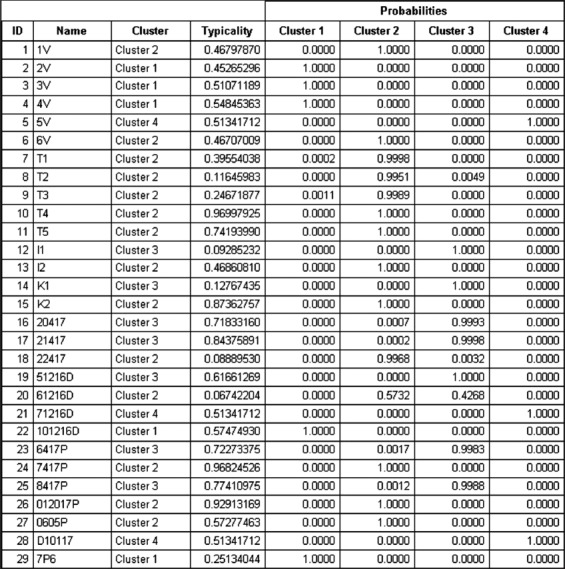
The ScreenClust output showing the four clusters of the unsupervised model with the typicality and probability of each sample to be in one of the four clusters.

**Figure-2:**
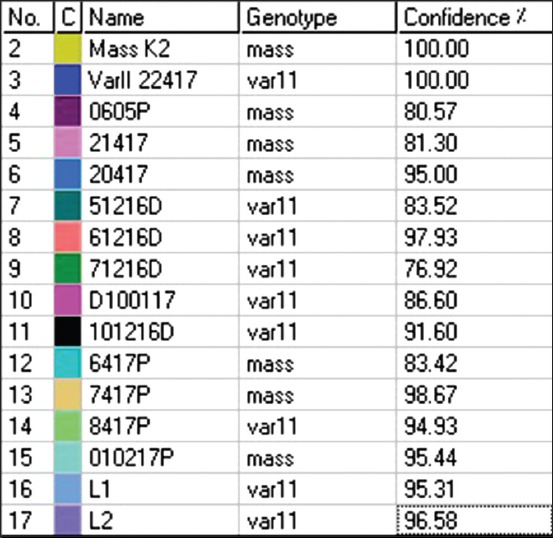
HRM genotype confidence percentages of selected IBV samples after normalization of the data.

**Figure-3:**
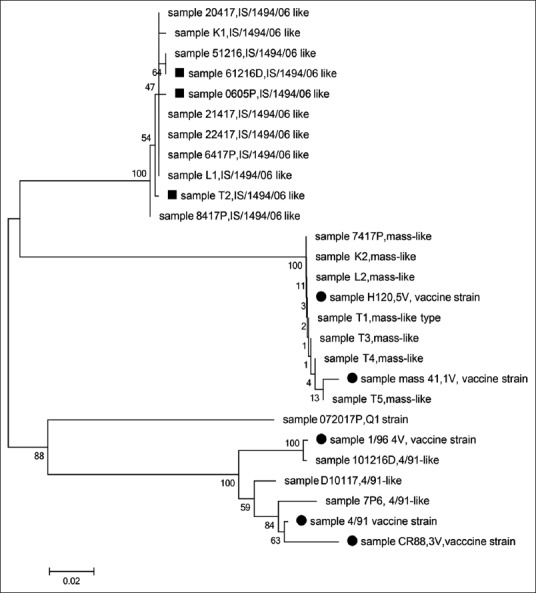
Phylogenetic tree analysis of the partial spike 1 gene sequences of IBV vaccine strains and field isolates. The solid circle is the IBV vaccine strains. The solid squares are the mismatched IBV (in HRM analysis, those isolates were clustered in cluster 2 (Mass-like type), but in sequencing, they were of IS/1494/06 like). The evolutionary history was inferred using the neighbor-joining method. The percentage of replicate trees in which the associated taxa clustered together in the bootstrap test (1000 replicates) is shown next to the branches. There were a total of 315 positions in the final dataset. Evolutionary analyses were conducted in MEGA6.

To detect the field and vaccine IBV strains, we used a nested RT-PCR assay to detect the S1 gene. Twenty-two out of the 23 samples were positive for the S1 gene after nested RT-PCR. Those 22 samples, along with the five IBV vaccine reference strains, were subjected to qRT-PCR targeting the S1 gene followed with HRM curve analysis. Table-3 summarizes the results for HRM and ScreenClust analysis as well as the sequencing of the 22 IBV field samples and 5 IBV vaccine samples (a total of 27 samples). The clusters are produced by the ScreenClust software into four clusters with the typicality and probability of each sample to be in one of the four clusters (Figure-1). Furthermore, the HRM GCPs of selected samples are shown in Figure-2. The sequencing of the partial S1 gene was carried out to confirm the results of the HRM assay (Figure-3). The sequencing of the partial S1 gene for all five IBV vaccine strains matched 100% with their identity. Out of the 22 field samples, three samples were found to be IBV 4/91-like strain on sequencing and on HRM analysis. Out of the 22 field samples, seven samples were confirmed with sequencing and HRM analysis to be of Mass-like strain. Eleven IBV field samples were found to be of IBV variant II-like (IS-1494-06) field strain (on HRM, 8/11 were also of variant II-like and 3/11 were of Mass like) and one field sample was a unique IBV strain (Q1 strain). The HRM assay divided the IBV vaccine and field strains into four different clusters.

**Table-3:** Correlation between the HRM melting temperature, the cluster numbers according to the ScreenClust software, and the sequencing results.

Number	Sample ID	HRM melting temp.	Screen plot cluster	Sequencing result
1	1V	78.24	CLUSTER 2	Mass
2	2V	78.36	CLUSTER 1	4\91
3	3V	77.79	CLUSTER 1	CR 88 variant strain very close to 4\91
4	4V	78.09	CLUSTER 1	1\96 very close to 4\91
5	5V	78.39	CLUSTER 2	H-120 very close to Mass
6	T1	78.81	CLUSTER 2	Mass
7	T2	78.39	CLUSTER 2 (borderline of cluster 2)	Variant II – mismatch
8	T3	78.36	CLUSTER 2	Mass
9	T4	78.36	CLUSTER 2	Mass
10	T5	78.36	CLUSTER 2	Mass
11	L1	78.90	CLUSTER 3	Variant II
12	L2	78.45	CLUSTER 2	Mass
13	K1	79.05	CLUSTER 3	Variant II
14	K2	78.39	CLUSTER 2	Mass
15	20417	78.90	CLUSTER 3	Variant II
16	21417	78.75	CLUSTER 3	Variant II
17	22417	78.60	CLUSTER 3	Variant II
18	51216D	78.81	CLUSTER 3	Variant II
19	61216D	78.75	CLUSTER 2 (borderline of cluster 3)	Variant II – mismatch
20	101216D	78.30	CLUSTER 1	4\91
21	6417P	78.30	CLUSTER 3	Variant II
22	7417P	78.51	CLUSTER 2	Mass
23	8417P	78.75	CLUSTER 3	Variant II
24	072017P	78.69	CLUSTER 4	Q1
25	0605P	78.45	CLUSTER 2	Variant II – mismatch
26	D10117	77.45	CLUSTER 1	4\91
27	7P6	77.49	CLUSTER 1	4\91

HRM: High-resolution melting

#### Cluster 1

The first cluster consisted of the IBV 4/91 and IBV 4/91-like strains. Three vaccines and three field isolates were located in this cluster. The IBV strain 4/91 can be found both as a vaccine and as a field strain and many IBV infections in different countries have been linked to 4/91 field strains. The IBV field samples (D10117, 101216D, and 7P6) belonged to cluster 1, representing the 4/91 and IBV 4/91-like strains, respectively. Based on HRM analysis, the melting temperature of the 101216D sample (78.30°C) was close to that of the 4V (1/96) strain (78.09°C), while the melting temperatures of the 7P6 sample (77.49°C) and the 3V (CR88 variant) strain (77.09°C) were similarly close. Despite the variation in melting temperatures between the vaccine and field strains in cluster 1, HRM and ScreenClust analysis were still able to identify the similarities between them by classifying them into the same cluster.

#### Cluster 2

The second cluster contained the IBV Mass and Mass-like strains. Two IBV vaccine strains were located in this cluster along with 10 field IBV isolates, three of which were found to be of a variant II-like (1S/1494/06-like) strains on sequencing (T2, 61216D and 0605P). While cluster 2, which includes IBV mass and mass like strains, has a closer range between the melting temperatures (78.24-78.51) with less variation up to 0.27. This indicates that the viruses of the field samples share close sequences to the Mass vaccine strain.

#### Cluster 3

The third cluster contained the IBV variant II-like (1S/1494/06-like) strains, and only field isolates were found in this cluster. This cluster has the highest difference in the melting temperature that reaches 0.75. Eight samples were of variant II-like (1S/1494/06 like) IBV strain representing 34% of the total IBV samples.

#### Cluster 4

The fourth and final cluster included one IBV field strain (072017), the latter of which was found to be Q1-like strain on sequencing.

## Discussion

HRM analysis has been used to detect and genotype fowl adenoviruses, avian influenza virus, and avian nephritis virus, among others [17,21,22]. The first published study to use HRM analysis to the type of IBV strains was carried out in Australia, where an HRM assay that targeted the 3’UTR gene was performed on local IBV vaccine and field strains [16]. Of the 17 IBV-positive cases, only 12 were typed correctly with matching HRM/3’UTR and S1 gene sequencing results [16]. As can be seen, our HRM assay can type any Mass or 4/91 IBV vaccine and field strain correctly to their corresponding cluster. However, a problem is only encountered when dealing with IBV variant II field strain. In such cases, we might need to confirm the results of the HRM assay by sequencing of the S1 gene.

Regarding cluster 3, the variant II-like (1S/1494/06-like) stain is an endemic IBV strain in Jordan that is involved in the respiratory, kidney, and reproductive tract problems [23,24]. Recently, the variant II-like (1S/1494/06-like) field strain has been found to be subject to multiple recombination events, which might explain the wide range in melting temperatures in cluster 3 [25].

Concerning cluster 4, sample 072017 was found to belong to the unique Q1 strain, which was first reported in China, in 1996, and then in Italy during a 2011 outbreak in broilers [26,27]. Even though the melting point for this sample is similar to those in other clusters, HRM and ScreenClust analysis were able to differentiate it into a separate cluster.

## Conclusion

Our qRT-PCR with HRM/spike gene assay was able to genotype all vaccine IBV strains and cluster them into separate clusters according to their distinct genotypes (Mass and Mass-like cluster, 4/91 and 4/91-like cluster). Most of the IBV field samples belonged to the variant II-like (1S/1494/06-like) strain. Those variant II-like (1S/1494/06-like) strains were all classified into one cluster expect for three samples that were clustered within the Mass and Mass-like IBV strains. The current qRT-PCR/HRM curve analysis was able to detect and rapidly identify novel and vaccine-related IBV strains which are confirmed by S1 gene nucleotide sequencing, making it a rapid and cost-effective tool.

## Authors’ Contributions

MA and OA designed the experiment. OA performed the technical assays. MA and MBA supervised the technical assays, MA and OA analyzed the results. MA and OA prepared the manuscript. All authors read and approved the final manuscript.
